# Supplementary material for: Inflammatory factors and risk of lung adenocarcinoma: a Mendelian randomization study mediated by blood metabolites
Source: Front Endocrinol (Lausanne). 2024 Aug 27;15:1446863. doi: 10.3389/fendo.2024.1446863 (PMC11384989; doi:10.3389/fendo.2024.1446863)
Supplement: Supplementary file 1 [file DataSheet1.zip › supplementary files/S2/GCST90200731/forest.pdf]

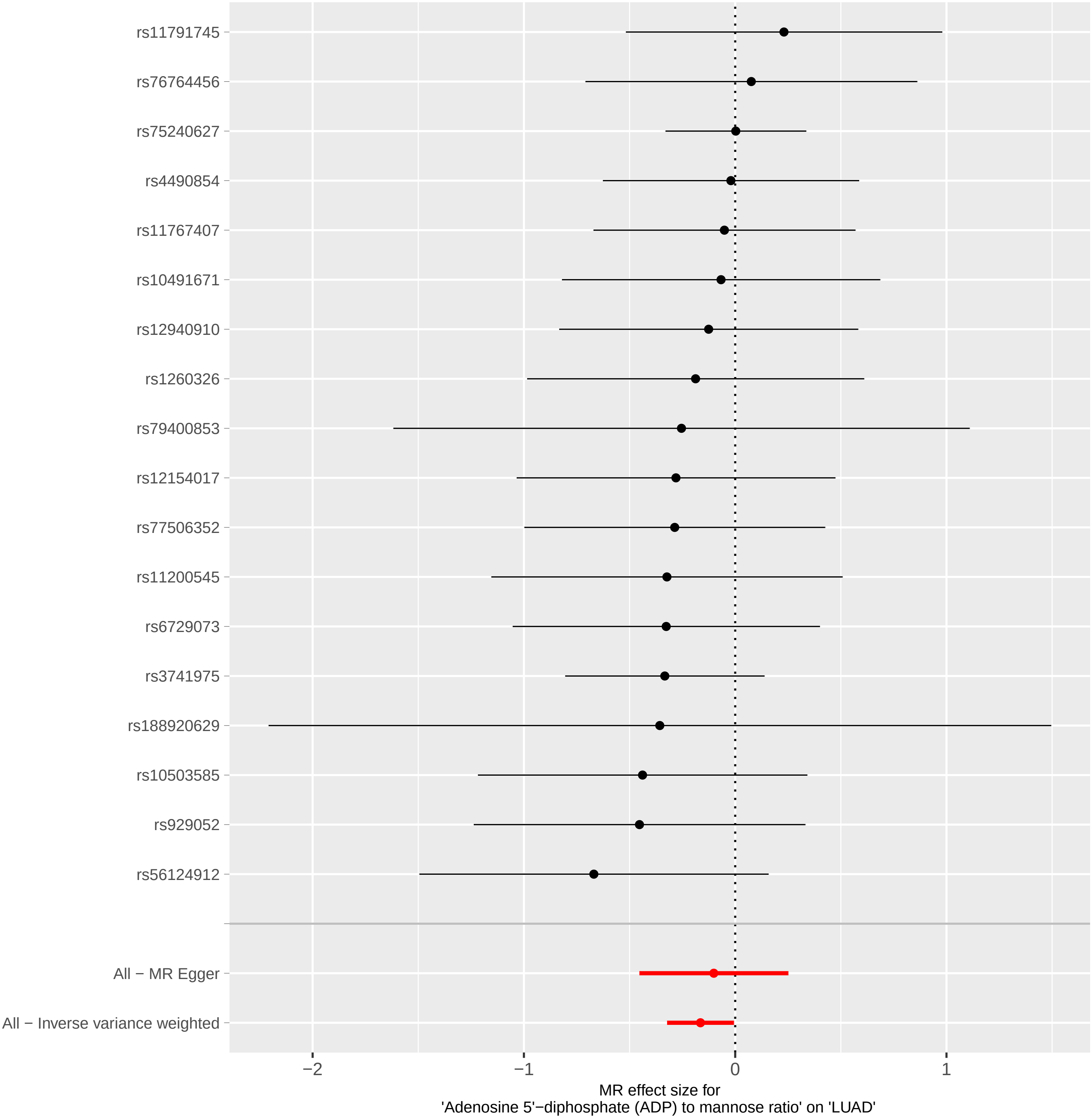
rs11791745
rs76764456
rs75240627
rs4490854
rs11767407
rs10491671
rs12940910
rs1260326
rs79400853
rs12154017
rs77506352
rs11200545
rs6729073
rs3741975
rs188920629
rs10503585
rs929052
rs56124912
All − MR Egger
All − Inverse variance weighted
−2
−1
0
1
MR effect size for
'Adenosine 5'−diphosphate (ADP) to mannose ratio' on 'LUAD'
